# Current Status of Prurigo Nodularis in Japan: A Retrospective Study Using a Health Insurance Claims Database †

**DOI:** 10.3390/jcm14061872

**Published:** 2025-03-11

**Authors:** Atsuyuki Igarashi, Takuo Yoshida, Yoshinori Sunaga, Hisakatsu Nawata, Kazuhiko Arima

**Affiliations:** 1 Igarashi Dermatology Higashigotanda, Tokyo 141-0022, Japan; 2 Medical Affairs, Sanofi K.K., Tokyo 163-1488, Japan

**Keywords:** claims analysis, evidence, health resources, Japan, prevalence, prurigo nodularis, retrospective study, therapy

## Abstract

**Background/Objectives:** Prurigo nodularis (PN) is associated with considerable disease burden. Limited information exists about the epidemiology, treatment patterns, and impact of PN. This retrospective study used Japanese health insurance claims data to investigate the prevalence and incidence of PN from 2006 to 2021. **Methods:** A cross-sectional study design was used to estimate prevalence and incidence longitudinally; a cohort design was used to assess comorbidities, treatment patterns, and healthcare resource utilization (HCRU). **Results:** Over the study period, data from 297,545 to 10,081,414 individuals were available annually; in 2020, 1946 individuals were diagnosed with PN. The prevalence and incidence of PN showed little variation over the study period; in 2021, the prevalence was 41 per 100,000 persons. Although there was a tendency for a higher prevalence of PN in childhood, the prevalence and incidence were similar in other age groups and were slightly higher in females. Inflammatory skin diseases and atopic diathesis were common comorbidities. The most prescribed treatments for adults with PN were topical steroids (78%), oral antihistamines (68%), and moisturizers (54%). Oral steroids, macrolides, and psychotropics were prescribed to >10% of patients. Individuals with PN who also had atopic dermatitis (AD) received higher cumulative doses of stronger potency topical steroids, local steroid injections, and oral steroids than those without concomitant AD. Additionally, HCRU was higher in individuals with AD. **Conclusions:** Although patients are usually treated according to the guidelines, there is an unmet need for more effective treatments for PN due to the frequent use of intensive and late-line treatments.

## 1. Introduction

Prurigo is a chronic inflammatory skin condition characterized by multiple, intensely pruritic papules or nodules [1,2]. The intense itching results in scratching, which sets up a vicious cycle of chronic itch followed by continual scratching [3]. The characteristic itch, plus the appearance of the lesions and bleeding in affected areas, result in considerable disease burden and a negative impact on quality of life [4,5,6,7].

Several types of prurigo have been recognized in Japan, including prurigo nodularis (PN) [1]. PN is associated with the presence of firm, symmetrically distributed nodules, which usually appear on the trunk or limbs [2]. The burden of disease among individuals with PN is reportedly higher than that of some other forms of inflammatory dermatosis [5]. However, the concept and classification of PN vary from country to country, and there is currently no well-established consensus on its pathoetiology and diagnosis, leading to ongoing clinical uncertainty over the condition and its management [1].

A few treatment algorithms for the management of PN are available [1,3,8]. These suggest a multimodal approach targeting any underlying cause and using various treatments, including topical therapies (e.g., moisturizers and emollients and phototherapy [ultraviolet B; UVB]) and systemic therapies (e.g., antihistamines, gabapentinoids [gabapentin, pregabalin], and immunosuppressants) [1,3,8]. Nevertheless, treatment satisfaction remains low [7], and many of the recommended medications were previously used off-label as, until recently, no treatments for PN had been approved by any regulatory body globally [8,9].

There are no data on epidemiology, treatment patterns, and healthcare resource utilization (HCRU) in individuals with PN including prurigo in Japan. Therefore, in this study, we aimed to investigate the prevalence, incidence, and treatment practices for PN in Japan using data from a prescription claims health insurance database. Moreover, evidence supports the existence of PN in two different forms—with and without atopic dermatitis (AD)—but there are no reported investigations based on large-scale data on the prevalence, incidence, and treatment practices of these forms [10,11]. The findings from this study may provide a better understanding of the real-world issues associated with PN and assist in identifying unmet medical needs in individuals with the condition.

## 2. Materials and Methods

### 2.1. Study Design and Objectives

This retrospective observational study (UMIN-CTR UMIN000050273) utilized information from the JMDC claims database (JMDC Inc., Tokyo, Japan).

The primary objective was to estimate the prevalence of PN in Japan by age, sex, and in individuals with/without AD and with/without atopic diathesis. Secondary objectives were to (i) estimate the incidence of PN; (ii) clarify the comorbidities and HCRU associated with PN and current treatments being prescribed for PN in general and PN in individuals with/without AD; (iii) investigate the prevalence, incidence, comorbidities, treatment, and HCRU associated with a wider category of prurigo (all types of prurigo, defined by International Classification of Diseases 10th revision [ICD-10] codes L28.1 and L28.2) by age, sex, and in individuals with/without AD and with/without atopic diathesis; and (iv) describe treatments for prurigo chronica multiformis (PCM) as reference information.

A cross-sectional design was used to estimate the prevalence and incidence of PN while a cohort design was used to assess comorbidities, treatment patterns, and HCRU. Data from 2006 to 2021 were used in the cross-sectional analysis, with the index month being the month of first diagnosis of PN during each year. For the cohort analysis, the data period was January 2019 to December 2021, with the index period being January to December 2020 and the index month defined as the month of first diagnosis of PN during the index period (Supplementary Figure S1). The baseline period was the 12 months prior to and including the index month, and the follow-up period was the 12 months after the index month.

As there are no clear diagnostic criteria for PN in Japan, and “prurigo” is often used interchangeably for referring to PN, the same methodology that was used to identify and investigate the data for PN was utilized to identify and investigate the data for the wider category of prurigo throughout this study for future reference.

Authors from Sanofi were involved with external authors in conceptualization, analysis, interpretation, drafting, revision, and approval for the final draft publication.

### 2.2. Ethical Considerations

This study did not involve the collection, use, or transmission of individually identifiable data; therefore, informed consent was not required. The study protocol was submitted to the Riverside Institutional Review Board (Sapporo, Japan) for review and approval. This study was conducted according to the guidelines for Good Pharmacovigilance Practice and Good Pharmacoepidemiology Practice issued by the International Society for Pharmacoepidemiology, the Declaration of Helsinki and its amendments, and applicable national guidelines, laws, and regulations.

### 2.3. Database

The JMDC database is a real-world, anonymized, Japanese insurance database which contains claims data from various health insurance societies and mainly includes individuals who have company health insurance and their families and does not include individuals who are self-employed, employed in agriculture or fisheries, or who are irregularly working or retired [12]. The JMDC database is suitable for the present clinical questions which we tried to address in this study (i.e., the trend in prevalence and current real-world treatment for the practice of PN) given its high patient follow-up rate and approximate representativeness in the Japanese population [12]. This database has also been utilized similarly for the study of other diseases [13,14]. The cumulative dataset includes information on inpatient, outpatient, and dispensing claims data for 7,779,860 insured Japanese people (as of December 2021). This represents approximately 8% of the total Japanese population and 34% of Japanese company employees and their family members who are members of health insurance associations in Japan.

The available data include age, sex, eligibility gained/lost date, and claims data including treatment, laboratory and diagnostic tests performed, duration of hospitalization, medication (Anatomical Therapeutic Chemical code, product/brand, daily dosage [for oral and intravenous medications], and duration), diagnosis (ICD-10).

### 2.4. Analysis Cohort

The study population consisted of individuals (the insured person or their family members) who were included in the JMDC claims database.

The PN group included individuals with PN specifically (ICD-10 code L28.1; disease name: nodular prurigo). The prurigo group included individuals with other prurigo types (ICD-10 code L28.1, L28.2), i.e., individuals with PN, and prurigo not otherwise specified (which includes PCM, Hebra’s prurigo, subacute prurigo, papular urticaria, acute prurigo, prurigo pigmentosa, chronic prurigo, and prurigo).

Individuals with at least one diagnosis for AD or for a wider category of atopic diathesis (i.e., AD, asthma, allergic rhinitis [including allergic rhinoconjunctivitis], or food allergy) in the 12 months before and after the index month were also identified and allocated to the AD or atopic diathesis cohorts, respectively.

The cross-sectional analysis included individuals who were diagnosed with PN during the 2005–2021 period, while the cohort analysis included those diagnosed with PN in 2020.

Prevalence was determined in individuals who had been enrolled in the JMDC database continuously during each analysis year (2006–2021). Incidence was determined in individuals who had enrolled continuously during each analysis year (2006–2021) and in the year prior to each analysis year and had no diagnosis of PN in the year prior to the index month.

Comorbidities, treatment, and HCRU were determined in individuals aged >15 years who were diagnosed with PN in 2020 and had 12 months’ continuous enrolment at the baseline period including the index month and ≥12 months’ continuous enrolment at the follow-up period after the index month. Individuals aged <15 years were excluded from the analysis because the pathophysiology of PN appears to differ between children and adults [15].

### 2.5. Endpoints

The primary endpoint was the prevalence of PN for each year between 2006 and 2021. A standardized prevalence for the total Japanese population was also calculated. In addition, the group-specific prevalence for each year was calculated for age at index date, sex, and the presence/absence of atopic diathesis or AD.

The secondary endpoints included the incidence of PN each year (overall and by age, sex, and the presence/absence of atopic diathesis and AD) and endpoints related to comorbidities (particularly those that have been commonly reported in surveys in other countries [16,17,18]), treatment patterns, and HCRU (see Supplementary Table S1 for further details). Because the JMDC database does not include information on the severity of PN, severity was estimated using the class of drug treatment as a proxy and by referring to a previous report on AD [19]; five severity levels (from most [4] to least [0] severe) were determined (see Supplementary Methods for definitions) [20].

### 2.6. Statistical Analysis

As this was an observational, descriptive study, no formal hypothesis was tested; therefore, no sample size calculation was required. Data from all individuals who met the inclusion criteria were included in the analysis. Missing dates were not replaced, and when creating data analysis sets, null information was complemented to zero.

The methods of calculating prevalence and incidence are summarized in the Supplementary Methods. Descriptive statistics were used to describe demographic and clinical characteristics (including comorbidities), treatment patterns, and HCRU. Numbers, means, 95% confidence intervals (CIs; Clopper–Pearson intervals), standard deviations (SDs), medians, interquartile range (IQR: first quartile [Q1]–third quartile [Q3]), and minimum and maximum were used to summarize continuous variables, and numbers and percentages were used for categorical variables.

Data analysis was performed using SAS version 9.4 (TS1M6) [SAS Institute, Cary, NC, USA].

## 3. Results

### 3.1. Study Population

The number of individuals in the JMDC database increased each year, from 297,545 in 2006 to 10,081,414 in 2021 (Supplementary Figure S2). In the cross-sectional analysis, the population for whom the prevalence of PN was determined ranged from 247,727 in 2006 to 7,779,860 in 2021, while the population for whom the incidence of PN was determined ranged from 224,955 in 2006 to 6,627,954 in 2021.

Of the 7,376,571 individuals in the index period of the cohort analysis (January–December 2020), 8588 met the inclusion criteria for the cohort analysis (Supplementary Figure S3). Of all 1946 individuals who were diagnosed with PN at the index month, 961 also had AD and 985 did not (Supplementary Figure S3). Those with PN at the index month had a mean ± SD age of 41.9 ± 13.5 years, with the most common age categories being those aged 40–49 years (28.8%) and 50–59 years (23.2%), although approximately one-fifth (20.6%) were aged 15–29 years; approximately half of the individuals (51.3%) were males (Table 1). Disease severity was Level 3 in 52.0% of individuals, while 18.1% had Level 4 severity and 14.3% had Level 0. Compared with the PN cohort, the mean ± SD age of the PN with AD cohort was slightly younger (39.9 ± 13.4 years) and in the PN without AD cohort was slightly older (43.9 ± 13.2 years); the most common age category was also 40–49 years in the PN with AD cohort and the PN without AD cohort (Table 1). The PN with AD cohort had more males (55.8%) than females (44.2%); conversely, the PN without AD cohort had more females (53.1%) than males (46.9%). More individuals in the PN with AD cohort had a disease of Level 3 (60.8%) or Level 4 (23.8%) severity than in the general PN or PN without AD cohorts. More individuals in the PN without AD cohort had Level 0 disease severity (22.8%) than in the other two cohorts (5.5–14.3%).

Individuals in the wider prurigo cohort (N = 8588) had a similar mean ± SD age (42.6 ± 14.0 years) and age category distribution to those in the PN cohort (Supplementary Table S2).

### 3.2. Prevalence and Incidence

**Figure 1:**
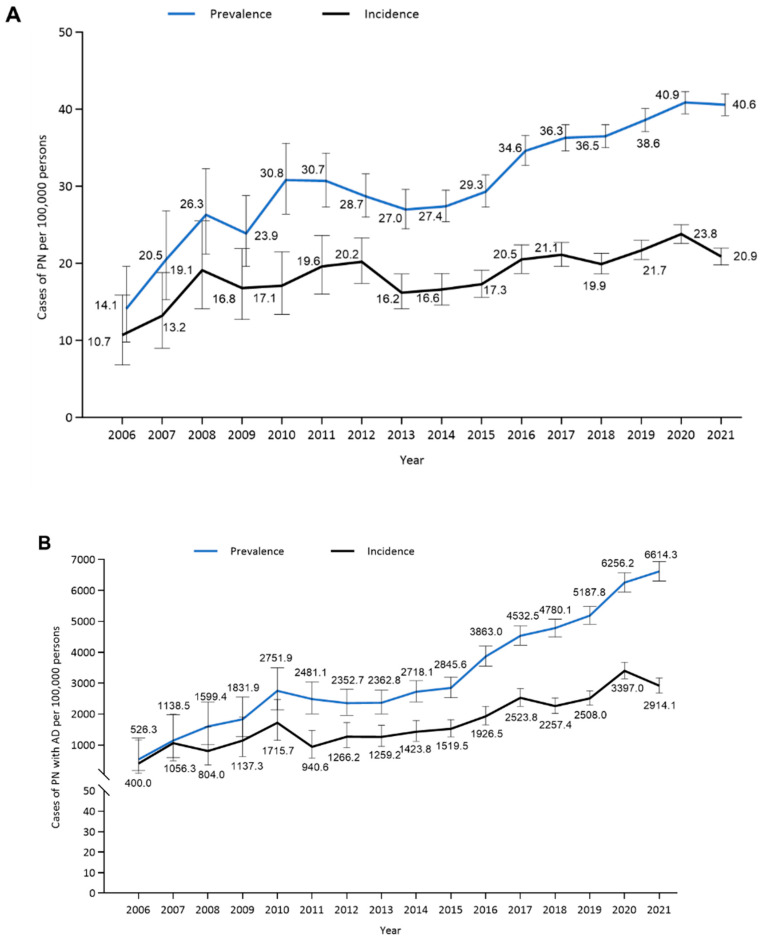

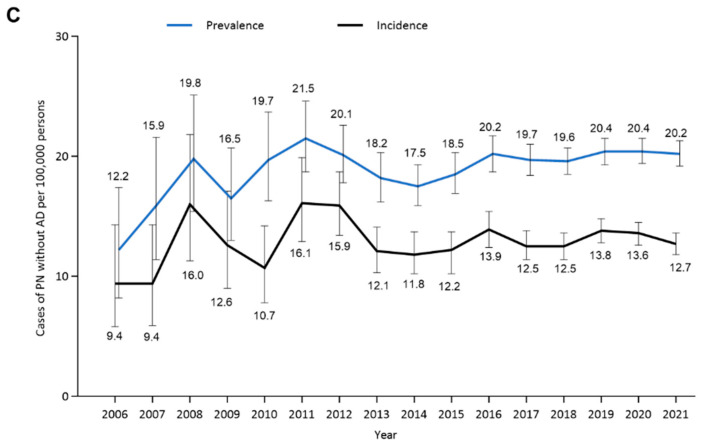
Prevalence and new onset incidence of prurigo nodularis (PN) among (**A**) the overall population, (**B**) those with atopic dermatitis (AD), and (**C**) those without AD, from 2006 to 2021. Error bars indicate 95% confidence intervals.

The prevalence of PN increased between 2006 and 2021, from 14 per 100,000 persons in 2006 to 41 per 100,000 persons in 2021; the incidence of new-onset PN increased from 11 per 100,000 persons in 2006 to 21 per 100,000 persons in 2021 (Figure 1A). When assessed based on the presence of comorbid AD, the prevalence of PN with AD increased substantially from 526 per 100,000 persons in 2006 to 6614 per 100,000 persons in 2021 (Figure 1B), whereas the increase in the prevalence of PN without AD was small (from 12 per 100,000 persons in 2006 to 20 per 100,000 persons in 2021; Figure 1C). Similar trends were observed with the incidence rates, with a markedly higher increase in the incidence of PN with AD observed between 2006 and 2021 (Figure 1B) compared with the incidence of PN without AD (Figure 1C).

Across the study period, the prevalence and incidence of PN were slightly higher in females than in males (Supplementary Figure S4A,B, respectively).

Individuals with AD were more likely than those without AD to have a diagnosis of PN (Figure 1B,C). In individuals with atopic diathesis (Supplementary Figure S5A,B), this trend was less strong than that observed in those with AD. Further, the overall prevalence and new onset incidence of PN among individuals with an AD or atopic diathesis comorbidity increased over the study period, with an increase of greater magnitude observed in individuals with AD than in those with atopic diathesis as a comorbidity. The prevalence and incidence of PN in individuals without atopic diathesis or AD did not change over the study period.

**Figure 2:**
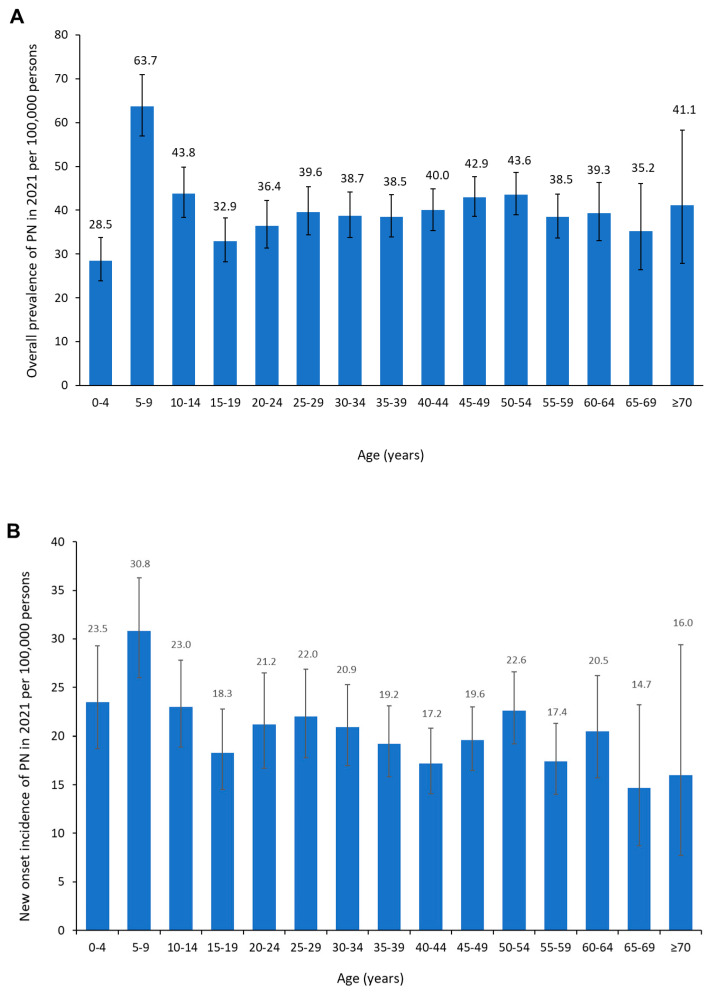
(**A**) Overall prevalence and (**B**) new onset incidence of prurigo nodularis in 2021 by age group. Error bars indicate 95% confidence intervals.

When considering the prevalence of PN in 2021 (41 per 100,000 persons), this value changed very little when adjusted for age and sex based on the demographic makeup of the total Japanese population (39 per 100,000 persons for PN in 2021). When compared by age groups, the overall prevalence (Figure 2A) and new onset incidence (Figure 2B) of PN in 2021 was highest in individuals aged 5–9 years (prevalence, 64 per 100,000 persons; incidence, 31 per 100,000 persons). Although the prevalence and incidence of PN were highest in these younger individuals, the parameters varied very little over the age range of 10 to ≥70 years (at approximately 33–44 and 15–23 per 100,000 persons, respectively).

The prevalence and incidence of prurigo are described in the Supplementary Results (including Supplementary Figure S4 [by sex], Supplementary Figure S5 [by presence or absence of AD or atopic diathesis], and Supplementary Figure S6 [by age group]). Trends for the prevalence and incidence of individual types of prurigo disease are described in Supplementary Figure S7, showing similar trends to those for PN.

### 3.3. Comorbidities

**Figure 3:**
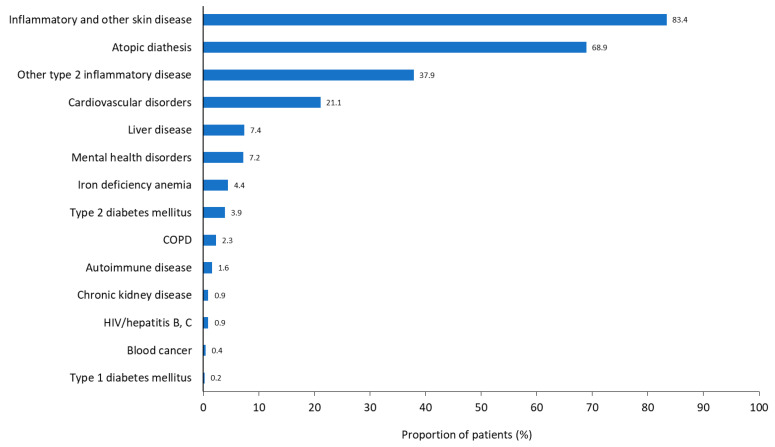
Overall prevalence of comorbidities in the prurigo nodularis cohort. Substance abuse was reported in <0.1% of the cohort. COPD, chronic obstructive pulmonary disease; HIV, human immunodeficiency virus.

Inflammatory skin diseases and atopic diathesis were the most common comorbidities in the PN cohort (83.4% and 68.9%, respectively); other comorbidities occurring in >3% of individuals in the PN cohort included other type 2 inflammatory diseases (37.9%), cardiovascular disorders (21.1%), liver disease (7.4%), mental health disorders (7.2%), iron deficiency anemia (4.4%), and type 2 diabetes mellitus (3.9%; Figure 3). The rates of these comorbidities did not differ markedly for the PN with AD and PN without AD cohorts (Supplementary Figure S8). Comorbidity data for all of these diseases in the prurigo cohort are shown in Supplementary Figure S9 and summarized in more detail in the Supplementary Results.

Inflammatory and other skin diseases occurring in ≥3% of individuals with PN were AD and xerosis cutis (43.5% and 55.4%, respectively; Supplementary Figure S10A). Atopic diathesis diseases observed in addition to AD in individuals with PN were allergic rhinitis and asthma (36.2% and 18.6%, respectively; Supplementary Figure S10B). Corresponding comorbidity data for these diseases in the prurigo cohort are shown in Supplementary Figure S10A,B, respectively, and summarized in more detail in the Supplementary Results.

### 3.4. Treatment

**Figure 4:**
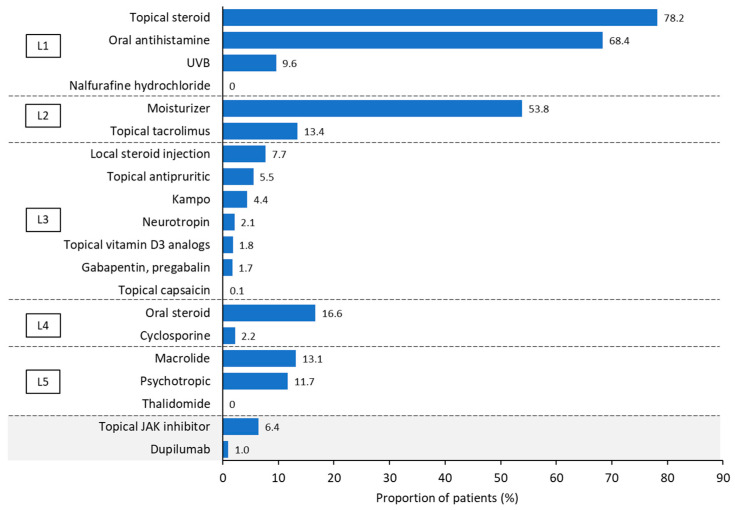
Treatment prescribed during the follow-up period in participants with prurigo nodularis. Treatments are categorized based on the Japanese Dermatological Association 2020 guidelines for prurigo [1]. Line 1 (L1) = topical steroid (TS) therapy ± antihistamines ± ultraviolet B phototherapy (with nalfurafine hydrochloride for individuals on hemodialysis, with chronic liver disease, or with other forms of prurigo); L2 = taper TS potency + moisturizer + tacrolimus ointment; L3 = TS therapy + adjunctive therapy (including combined used of local steroid injection, topical heparinoid [occlusive application], vitamin D3 analogs, tacrolimus ointment, antipruritic ointment, capsicum ointment, liquid nitrogen, neurotropin [extracted fluid from the inflamed skin of rabbits inoculated with vaccinia virus], reserpine, gabapentin/pregabalin, or traditional Chinese medicine); L4 = TS therapy + systemic steroid therapy or cyclosporine; or L5 = antibiotics (macrolides), anti-anxiety agents, or thalidomide. The treatments in the grey box at the bottom of the figure are not included in the guidelines. JAK, Janus kinase; UVB, ultraviolet B.

The most commonly prescribed types of treatment in the PN cohort were those that are recommended as early-line treatment for chronic prurigo [1,8], namely topical steroids (78.2%), oral antihistamines (68.4%), and moisturizers (53.8%; Figure 4). Treatments prescribed for the PN with AD and PN without AD cohorts are shown in Supplementary Figure S11.

Around 70% of individuals in the PN cohort were prescribed very strong potency steroids (70.9%); the proportion was higher for the PN with AD cohort (76.8%) and lower for the PN without AD cohort (62.9%; Table 2). Higher cumulative doses of the strongest, very strong, strong, and medium potency topical steroids; local steroid injections; and oral steroids were prescribed to individuals in the PN with AD cohort compared with those in the PN without AD cohort (Table 2). Moreover, the duration of oral steroid treatment was longer for individuals in the PN with AD cohort compared with those in the PN without AD cohort, whereas daily doses of oral steroids were similar for both study cohorts (Table 2). The mean ± SD proportion of days covered with oral steroids was longer for the PN with AD cohort compared with the PN without AD cohort (17.2 ± 23.7 vs. 11.2 ± 19.2 days, respectively).

Further, a higher proportion of individuals in the PN with AD cohort were prescribed cyclosporine compared with individuals in the PN and those in the PN without AD cohorts (4.3% vs. 2.2% and 0.1%, respectively; Figure 4 and Supplementary Figure S11). The results by the presence or absence of an atopic predisposition showed similar trends to those with or without AD (Supplementary Table S3).

Details of the treatments prescribed in the prurigo cohort and PCM cohort, which was investigated as a reference, are provided in the Supplementary Results (including Supplementary Figure S12 and Supplementary Table S3). Treatment in the PCM cohort was of a similar level to the more comprehensive treatment prescribed in the PN with AD cohort (Supplementary Table S3).

### 3.5. Healthcare Resource Utilization

Over two-thirds (71.2%) of individuals with PN required outpatient visits, which took a median of five (IQR 2–9) days per year; 9.5% were hospitalized, for a median of six (IQR 3–12) days per year (Table 3). A total of 78.7% of individuals with PN were prescribed medications associated with their disease, and 5.7% underwent clinical laboratory tests. In comparison, the proportion of people making outpatient visits, median (IQR) number of days per year spent on outpatient visits, and prescriptions for medications associated with their disease were higher for the PN with AD cohort and lower for the PN without AD cohort (Table 3).

Values for the prurigo cohort (Supplementary Table S4) were generally similar to those reported for the PN without AD cohort.

The results by the presence or absence of an atopic predisposition showed similar trends to those with or without AD (Supplementary Figure S13).

## 4. Discussion

This retrospective database study found that the prevalence of PN in Japan was 14 per 100,000 persons in 2006 and 41 per 100,000 persons in 2021. The prevalence increased somewhat from 2006 to 2016, but there was little variation over the rest of the study period. In 2021, the impact of COVID-19 might be considered when the incidence (new diagnosis of PN) is dropping sharply, as most outpatient visits suddenly decreased due to the pandemic [21,22], and the age/sex-adjusted prevalence of PN was 39 per 100,000 persons.

Patients with PN are more likely to be black or Asian compared with white patients [23,24]. However, the prevalence of PN found in our study (0.04%) was somewhat lower than that reported in studies from other countries. For example, an analysis of a United States (US) insurance claims database found a PN prevalence of 72 per 100,000 persons (0.072%) [25], while a German insurance database analysis reported a prevalence of 0.1% [17]. There are several possible reasons for this apparent variation in PN prevalence between our study and those from other countries, including differences between countries in the classification of pruritic rashes and in the awareness of PN [26], differences in the type of database used (e.g., our database included individuals aged 0–70 years old (average 41.9 years old) who were enrolled in a health insurance association, while the US database included individuals 18–64 years only (average 50.9 years old)), and international differences in insurance systems.

Data from this study indicate that PN was most prevalent in younger (aged <10 years) individuals. This is in contrast to the findings of other reports, which found a lower incidence rate of the condition in childhood [27]. The higher prevalence/incidence in childhood in our study suggests that acute prurigo, such as that arising from insect bites [15], is likely to be diagnosed and billed by clinicians in insurance claims as PN in Japan; however, this warrants further investigation.

The prevalence of PN was slightly higher for females than males; this difference was more marked for prurigo and is consistent with some reports that have indicated that females are more likely to experience prurigo than males [28,29], but not with others that have shown a higher prevalence in males [27]. Further, other studies, unlike ours, have reported a sex difference in the prevalence of PN, either higher in females than males [17,25,29] or higher in males than females [18]. The difference in results might be related to ethnic differences among cohorts from different countries [23,24] and the difference in the proportion of AD co-occurrence found in this study.

PN occurred more frequently in individuals with AD compared with those without AD. While the prevalence and incidence of PN did not increase markedly over the study period, the prevalence and incidence of PN in combination with AD did increase over time. Further, the prevalence and incidence of PN were higher in individuals with comorbid AD than in those with a more general atopic predisposition. These findings suggest that, of the atopic predispositions, AD is the most likely to be associated with the development of PN. Inflammatory skin diseases and atopic diathesis were the most common comorbidities in individuals with PN. Comorbid AD has been noted among individuals with PN in other studies, including those that assessed cohorts in Germany (15% of adults with PN had AD) [17] and the US (11% of adults with PN had AD) [23]; however, the prevalence of comorbid AD appeared to be generally higher in our Japanese cohort (e.g., AD in 44% of individuals). The prevalence of comorbid AD in our study was more consistent with that found in another study of Japanese individuals with PN (29%) [7].

In this study, individuals with PN generally received treatments as per the clinical practice guidelines for prurigo from the Japanese Dermatological Association (Supplementary Figure S14) [1], with over two-thirds prescribed topical steroids and antihistamines and approximately 50% prescribed moisturizers. However, the use of UVB, which is recommended by the Japanese Dermatological Association as the next step after topical steroids (Supplementary Figure S14) [1] and other therapeutic agents such as Line 3 drugs were limited, which has also been observed in a survey conducted in Japan [30]. Further, oral steroids, which are not recommended in the European guidelines for chronic prurigo, were used more frequently (in approximately 17% of individuals with PN) than several guideline-recommended earlier-line treatments (<8% for treatments such as local steroid injection, topical antipruritic, and topical vitamin D3 analogs). This may be related to a perceived lack of effectiveness of the currently recommended early lines of therapy, indicating an unmet need for more effective treatments. We hope such patients will be optimally managed with emerging medications such as dupilumab [31] and nemolizumab [32]. In addition, lack of efficacy [33], safety concerns when used in middle-aged and older patients who often have pruritus, and the burden of hospital visits for the administration of some drugs make continuous treatment difficult [34].

The presence of comorbid AD or atopic diathesis was associated with the prescription of more intensive treatment; this is to be expected given the likely cumulative nature of symptoms and their impact on individuals with more than one disorder [7].

Regarding HCRU, there were some differences between patients who had PN with and without atopic diathesis or AD, indicating greater HCRU in those with an atopic comorbidity. It is thought that HCRU is higher in individuals with this comorbidity because the disease burden is also higher [7].

Our study has a number of limitations that need to be considered when interpreting the results. Firstly, the diagnosis name was based on the disease name stated in the claims database, and PN was extracted using the ICD10 code; this may have differed from the actual diagnosis and may not have been correctly classified by the type of prurigo. Secondly, in the claims database, diseases are not linked to drug prescription data; therefore, some drugs (particularly steroids or antihistamines) may have been prescribed for other treatment purposes, and the prescribed drugs may not have been taken as described. It may cause misclassification. Thirdly, the JMDC database is derived from health insurance claims and consists of people who work for companies and their family members. Therefore, the data may be biased toward urban centers where many companies are located, and the findings should be interpreted in terms of generalizability. In addition, we must account for differences in healthcare systems, diagnostic criteria, and data collection methods when we compare with international data. Fourthly, in the JMDC database, the number of individuals aged >65 years was small, and those aged >75 years were not included because membership in insurance associations is terminated at age 75 years, after which Japanese individuals are covered by national health insurance. It may bias prevalence and incidence estimates. Fifthly, there are several drugs, tests, and treatments that are effective in PN but only have insurance coverage in Japan for AD. Therefore, when these are used for PN, they may be billed by insurance companies for the indication of AD, and thus the number of patients with AD or AD complications in this study may have been overestimated. Finally, as data on the use of over-the-counter drugs were not collected, the effects of these drugs were not assessed. Also, because disease severity cannot be obtained from claims data, the intensity of prescribed treatment was used as a surrogate for disease severity. It may cause a potential bias due to unobserved data.

## 5. Conclusions

This is the first study to report the prevalence, incidence, and treatment of PN in Japan using the JMDC insurance claims database. The most recent data (2021) indicate a prevalence of PN of 41 per 100,000 persons and an incidence of 21 per 100,000 persons. Individuals were generally treated according to guidelines, but the frequent use of intensive and late-line treatments suggests an unmet need for more effective treatments for PN.

## Figures and Tables

**Table 1:** Demographic and clinical characteristics of individuals aged >15 years diagnosed with prurigo nodularis and with and without atopic dermatitis who were included in the cohort analysis.

Characteristic	PN Cohorts		
	PN N = 1946	PN with AD N = 961	PN Without AD N = 985
Age at index month, mean ± SD, years	41.9 ± 13.5	39.9 ± 13.4	43.9 ± 13.2
Age category at index month, n (%)			
15–29 years	401 (20.6)	244 (25.4)	157 (15.9)
30–39 years	367 (18.9)	193 (20.1)	174 (17.7)
40–49 years	560 (28.8)	270 (28.1)	290 (29.4)
50–59 years	452 (23.2)	200 (20.8)	252 (25.6)
60–69 years	139 (7.1)	45 (4.7)	94 (9.5)
≥70 years	27 (1.4)	9 (0.9)	18 (1.8)
Sex, male, n (%)	998 (51.3)	536 (55.8)	462 (46.9)
Disease severity level, n (%) †			
Level 4	353 (18.1)	229 (23.8)	124 (12.6)
Level 3	1021 (52.0)	584 (60.8)	428 (43.5)
Level 2	167 (8.6)	61 (6.3)	106 (10.8)
Level 1	136 (7.0)	34 (3.5)	102 (10.4)
Level 0	278 (14.3)	53 (5.5)	225 (22.8)

† Disease severity was based on prescribed treatments—Level 4: systemic treatment including oral cyclosporine A, OS regardless of TS use; Level 3: topical treatment (high potency) including TS (‘strongest’ and ‘very strong’ classes, defined according to the JDA 2016 guidelines [20]); Level 2: topical treatment (low-to-medium potency) including TS (‘strong’, ‘medium’, and ‘weak’ classes, defined according to the JDA 2016 guidelines [20]); Level 1: treated with drugs other than cyclosporine A, OS, or TS; Level 0: not treated with studied treatments. AD, atopic dermatitis; JDA, Japanese Dermatological Association; OS, oral steroids; PN, prurigo nodularis; SD, standard deviation; TS, topical steroids.

**Table 2:** Prescribing patterns of steroids in individuals with prurigo nodularis, with and without atopic dermatitis.

Item	PN Cohorts		
	PN N = 1946	PN with AD N = 961	PN Without AD N = 985
Topical steroids, n (%)	1521 (78.2)	872 (90.7)	649 (65.9)
Steroid potency †			
Strongest	718 (47.2)	454 (52.1)	264 (40.7)
Very strong	1078 (70.9)	670 (76.8)	408 (62.9)
Strong	691 (45.4)	445 (51.0)	246 (37.9)
Medium	527 (34.6)	378 (43.3)	149 (23.0)
Weak	7 (0.5)	5 (0.6)	2 (0.3)
Patch	241 (15.8)	146 (16.7)	95 (14.6)
Cumulative dose, g/year, mean ± SD †			
Strongest	154.6 ± 214.4	178.6 ± 226.1	113.2 ± 186.0
Very strong	220.3 ± 307.6	285.1 ± 331.7	113.8 ± 226.3
Strong	109.8 ± 183.5	142.8 ± 208.2	50.1 ± 103.7
Medium	47.5 ± 97.1	54.5 ± 107.0	29.6 ± 62.4
Weak	49.2 ± 55.0	42.9 ± 53.5	64.9 ± 77.6
Local steroid injection, n (%)	150 (7.7)	69 (7.2)	81 (8.2)
Cumulative dose, g/year, mean ± SD	306.0 ± 408.8	426.4 ± 455.2	143.6 ± 259.3
Oral steroids, n (%)	323 (16.6)	200 (20.8)	123 (12.5)
Cumulative dose, mg/year, mean ± SD	314.0 ± 558.7	372.6 ± 637.5	218.5 ± 382.4
Daily dose, mg, mean ± SD	9.6 ± 11.1	9.7 ± 11.6	9.4 ± 10.4
Prescription duration, days, mean ± SD	54.4 ± 81.5	62.7 ± 86.8	40.9 ± 70.3

† Potency of steroids was defined according to the JDA 2016 guidelines [20]. AD, atopic dermatitis; JDA, Japanese Dermatological Association; PN, prurigo nodularis; SD, standard deviation.

**Table 3:** Healthcare resource utilization in individuals aged > 15 years diagnosed with prurigo nodularis and included in the cohort analysis.

Item	PN N = 1946	PN with AD N = 961	PN Without AD N = 985
Outpatient visits, n (%)	1386 (71.2)	806 (83.9)	580 (58.9)
Median (Q1–Q3), days/year	5 (2–9)	6 (3–11)	3 (1–7)
Range (min, max)	1, 94	1, 94	1, 78
Hospitalizations, n (%)	184 (9.5)	86 (8.9)	98 (9.9)
Median (Q1–Q3), days/year	6 (3–12)	5 (3–13)	7 (4–10)
Range (min, max)	1, 75	1, 69	1, 75
Clinical laboratory tests, n (%)	110 (5.7)	89 (9.3)	21 (2.1)
Median (Q1–Q3), times/year	1 (1–2)	1 (1–2)	1 (1–1)
Range (min, max)	1, 8	1, 8	1, 7
Prescriptions for PN-associated medications, n (%)	1532 (78.7)	874 (90.9)	658 (66.8)
Median (Q1–Q3), times/year	4 (2–8)	6 (3–10)	3 (1–5)
Range (min, max)	1, 62	1, 62	1, 38

AD, atopic dermatitis; max, maximum; min, minimum; PN, prurigo nodularis; Q, quartile.

## Data Availability

Qualified researchers may request access to patient-level data and related documents (including, e.g., the clinical study report, study protocol with any amendments, blank case report form, statistical analysis plan, and dataset specifications). Patient-level data will be anonymized, and study documents will be redacted to protect the privacy of trial participants. Further details of Sanofi’s data sharing criteria, eligible studies, and process for requesting access can be found at https://www.vivli.org/. Access date: 5 March 2025.

## Supplementary Materials

The following supporting information can be downloaded at: https://www.mdpi.com/article/10.3390/jcm14061872/s1.
