# Supplementary material for: Current Status of Prurigo Nodularis in Japan: A Retrospective Study Using a Health Insurance Claims Database
Source: J Clin Med. 2025 Mar 11;14(6):1872. doi: 10.3390/jcm14061872 (PMC11943442; doi:10.3390/jcm14061872)
Supplement: Supplementary file 1 [file jcm-14-01872-s001.zip › jcm-3453887-supplementary.pdf]

Supporting Information

Supplementary Methods[a]

Severity of prurigo nodularis (PN): the JMDC database does not include information on the severity of PN; therefore, disease severity was estimated based on the class of drugs used and referring to a previous report on atopic dermatitis (AD).[1] Five severity levels were determined – Level 4 (most severe): systemic treatment including oral cyclosporine A, oral steroids (OS) regardless of topical steroid (TS) use; Level 3: topical treatment (high potency) including TS ('strongest' and 'very strong' classes, defined according to the Japanese Dermatological Association [JDA] 2016 guidelines[2]); Level 2: topical treatment (low-to-medium potency) including TS ('strong', 'medium' and 'weak' classes, defined according to the JDA 2016 guidelines[2]); Level 1: treated with drugs other than cyclosporine A, OS, or TS; Level 0 (least severe): not treated with studied treatments.

Calculation of PN prevalence: the crude and group-specific prevalence proportion and number of individuals by each PN cohort were calculated for each index year in the total population and separately by age groups (0–4, 5–9, 10–14, 15–19, 20–24, 25–29, 30–34, 35–39, 40–44, 45–49, 50–54, 55–59, 60–64, 65–69, and ≥70 years), sex (male and female), presence/absence of AD, and presence/absence atopic diathesis as follows:

Prevalence (per 100,000 persons) = (number of individuals in each cohort) / (entire population in the JMDC database) × 100,000

Expected number in each cohort = prevalence proportion of each cohort × Japanese population in each index year.

Age and sex standardized prevalence proportions for each cohort were also calculated directly using the Japanese national population for each associated year.[3] In addition, group standardized prevalence proportion was calculated by age group and sex, age standardized prevalence was adjusted by sex, and vice versa. To adjust for the fluctuation of age and sex proportion in the cohorts, standardized proportions were calculated from the assumption that prevalence is constant for the same sex and age as follows:

Age and sex standardized prevalence proportion (%)

$$= \frac{\left\{\sum_{i=x}^{n}\left(N'_{\text{jmdc male i}} \times \frac{N_{\text{japan male i}}}{N_{\text{jmdc male i}}}\right) + \sum_{i=x}^{n}\left(N'_{\text{jmdc female i}} \times \frac{N_{\text{japan female i}}}{N_{\text{jmdc female i}}}\right)\right\}}{\sum_{i=x}^{n} N_{\text{japan male i}} + \sum_{i=x}^{n} N_{\text{japan female i}}}$$

Where: N japan male/female = male/female Japanese population in each index year; N jmdc male/female = male/female entire population in JMDC database; N’ jmdc male/female = number of male/female individuals in each cohort in the JMDC database; i = age

The ranges of age were changed appropriately for prevalence proportion by age groups.

Calculation of PN incidence: the incidence was calculated as follows:

Incidence (per 100,000 person-years) = (number of newly diagnosed individuals) / (entire population in JMDC database × sum of observation period (year)) × 100,000

Where: newly diagnosed individuals = those newly diagnosed with PN; entire population in JMDC database = those in the JMDC database population with no diagnosis of PN in the 12 months before the index month; observation period = from index month to the end of the follow-up period.

[a]The same methodology was used to determine the severity and calculate the prevalence and incidence of the wider disease category of prurigo.

Supplementary Results

Prurigo

Prevalence and incidence

The prevalence of prurigo in 2021 was 175 per 100,000 persons (Supplementary Figure S5). The prevalence increased between 2006 and 2016 but varied little from 2016 to 2021. There was little change in the incidence over time.

Across the study period, the prevalence and incidence of prurigo were higher in females (range, 159–196 and 137–132 per 100,000 persons, respectively) than in males (106–159 and 83–99 per 100,000 persons, respectively; Supplementary Figure S4A and S4B).

Individuals with AD were more likely than those without AD to have a diagnosis of prurigo (Supplementary Figure 1B and 1C). Similarly, individuals with atopic diathesis were more likely than those without atopic diathesis to have a diagnosis of prurigo (Supplementary Figure S5A and S5B). Further, the overall prevalence and new onset incidence of prurigo among individuals with an atopic diathesis or AD comorbidity increased over the study period, with an increase of greater magnitude observed in individuals with AD than in those with atopic diathesis as a

comorbidity. The prevalence and incidence of prurigo in individuals without atopic diathesis or AD did not change over the study period.

When considering the prevalence of prurigo in 2021 (175 per 100,000 persons), this value changed very little when adjusted for age and sex based on the demographic makeup of the total Japanese population (181 per 100,000 persons in 2021).

The overall prevalence (Supplementary Figure S6A) and new onset incidence (Supplementary Figure S6B) of prurigo in 2021 was highest in individuals aged 5–9 years (prevalence, 297 per 100,000 persons; incidence, 204 per 100,000 persons). The prevalence of prurigo decreased and then plateaued among individuals aged 10–40 years, before increasing again among older individuals, such that prevalence was 236 per 100,000 in those aged ≥70 years. A similar pattern was seen for the incidence of prurigo, although the plateau in incidence lasted until approximately 65 years, before rising to 141 per 100,000 persons in individuals aged ≥70 years.

The prevalence and incidence of individual types of prurigo disease are shown in Supplementary Figure S7A and S7B, respectively. The most common disease types (ICD-10 code L28.2) were prurigo and acute prurigo. Although there was some variation in prevalence and incidence of these individual prurigo types over time, no consistent change over the study period was seen.

Comorbidities

Inflammatory skin diseases and atopic diathesis were the most common comorbidities in individuals with prurigo (72.4% and 58.1%, respectively; Supplementary Figure S9). Other comorbidities occurring in >3% of individuals with prurigo were other type 2 inflammatory diseases (38.3%), cardiovascular disorders (22.0%), liver disease (7.9%), mental health

disorders (7.7%), iron deficiency anemia (5.1%), and type 2 diabetes mellitus (3.6%).

Inflammatory and other skin diseases occurring in ≥3% of individuals with prurigo were AD and xerosis cutis (27.9% and 43.7%, respectively; Supplementary Figure S10A). Atopic diathesis diseases observed in addition to AD in individuals with prurigo were allergic rhinitis (36.9%) and asthma (14.2%; Supplementary Figure S10B).

Treatment

The most commonly prescribed types of treatment in individuals with prurigo were those that are recommended as early-line treatment for prurigo,[4,5] namely topical steroids (67.6%), oral antihistamines (62.2%), and moisturizers (43.9%) (Supplementary Figure S12). These proportions were generally similar to those reported for individuals with PN without AD, and lower than the proportions of individuals prescribed these treatments in the PN and PN with AD cohorts (Figure 4, Supplementary Table S3). Moreover, the proportion of individuals with prurigo who were prescribed oral steroids was similar to the proportions of individuals with PN but without AD and those in the general PN cohort (15.0% vs 12.5% and 16.6%, respectively), but lower than the proportion of individuals in the PN with AD cohort who received this treatment (20.8%) (Figure 4, Supplementary Table S3). Steroid potencies prescribed to individuals with prurigo were similar to those prescribed to individuals in the PN without AD cohort, and lower than steroid potencies prescribed to the general PN and PN with AD cohorts (Supplementary Table S3; Table 2). In addition, individuals with prurigo were prescribed lower cumulative doses of topical steroids and local steroid injections, and had a shorter duration of oral steroid treatment than individuals in the general PN and PN with AD cohorts. Conversely,

individuals with prurigo received higher cumulative doses of topical steroids and local steroid injections, and had a longer duration of oral steroid treatment than individuals in the PN without AD cohort.

Healthcare resource utilization

Around half (58.3%) of individuals with prurigo had an outpatient visit in 2020 (Supplementary Table S4). The prurigo cohort was not markedly different from the other PN cohorts regarding the frequency at which medications were prescribed, the incidence or duration of hospitalizations, and incidence or number of clinical laboratory tests.

Supplementary Tables

Supplementary Table S1. Primary and secondary endpoints.[a]

**Primary endpoint**
Prevalence of PN (2006–2021)
Overall[b]
By age at index (0–4, 5–9, 10–14, 15–19, 20–24, 25–29, 30–34, 35–39, 40–44, 45–49, 50–54, 55–59, 60–64, 65–69, ≥70 years)
By sex (male, female)
By presence/absence of AD or atopic diathesis
**Secondary endpoints**
Incidence of PN (2006–2021)
Overall
By age at index (0–4, 5–9, 10–14, 15–19, 20–24, 25–29, 30–34, 35–39, 40–44, 45–49, 50–54, 55–59, 60–64, 65–69, ≥70 years)
By sex (male, female)
By presence/absence of AD or atopic diathesis
Comorbidities
Inflammatory and other skin disease,[c] mental health disorders,[d] endocrine disorders,[e] cardiovascular disorders,[f] chronic kidney disease, COPD, HIV, hepatitis B, hepatitis C, malignancy,[g] autoimmune disease,[h] substance abuse, iron deficiency anemia, atopic diathesis, other disease with type 2 inflammation,[i] liver disease
Treatment

Type of drug (TS, local [intralesional] injection of steroid, antihistamine, UVB [phototherapy], immunosuppressant [OS, non-steroidal oral immunosuppressant], topical antipruritic, moisturizer, topical vitamin D3 analogs, atopic dermatitis treatment [dupilumab, JAK inhibitor, PDE4 inhibitor])
HCRU
Outpatient visits associated with PN (days/year)
Hospitalizations (days/year)
Clinical laboratory tests (times/year)
Prescription for PN-associated medications (times/year)

[a]The same endpoints/methodological approaches were used to interrogate the data for PN and for the wider disease category of prurigo.

[b]Overall prevalence was calculated from the JMDC database and standardized to the entire Japanese population.

[c]AD, psoriasis, lichen planus, dermatitis herpetiformis, xerosis cutis, excoriation disorder, keratoacanthoma, bullous pemphigoid, linear IgA disease.

[d]Mood disorders, depression, anxiety, eating disorders, self-harm, attention-deficit/hyperactivity disorder, schizophrenia.

[e]Type 1 or 2 diabetes mellitus.

[f]Hypertension, hyperlipidemia, obesity, heart failure, cerebrovascular disease, coronary heart disease.

[g]Cutaneous T-cell lymphoma, mycosis fungoides, non-Hodgkin and Hodgkin's lymphoma, multiple myeloma.

[h]Celiac disease, inflammatory bowel disease (Crohn's disease, ulcerative colitis), Hashimoto thyroiditis.

[i]Chronic rhinosinusitis with nasal polyp, conjunctivitis, urticaria, allergic contact dermatitis, drug allergy, metal allergy, eosinophilic esophagitis.

AD, atopic dermatitis; COPD, chronic obstructive pulmonary disease; HCRU, healthcare resource utilization; HIV, human immunodeficiency virus; IgA, immunoglobulin A; JAK, Janus kinase; OS, oral steroid; PDE4, phosphodiesterase-4; PN, prurigo nodularis; TS, topical steroid; UVB, ultraviolet B.

Supplementary Table S2. Demographic and clinical characteristics of individuals aged >15 years diagnosed with prurigo all class and included in the cohort analysis.

**Characteristic**	**N=8,588**
Age at index month, mean ± SD, years	42.6 ± 14.0
Age category at index month, n (%)	
15–29 years	1,748 (20.4)
30–39 years	1,564 (18.2)
40–49 years	2,261 (26.3)
50–59 years	2,084 (24.3)
60–69 years	793 (9.2)
≥70 years	138 (1.6)
Sex, male, n (%)	4,098 (47.7)
Disease severity level, n (%)†	
Level 4	1,372 (16.0)
Level 3	3,791 (44.1)
Level 2	761 (8.9)
Level 1	841 (9.8)
Level 0	1,823 (21.2)

†Disease severity was based on prescribed treatments – Level 4: systemic treatment including oral cyclosporine A, OS regardless of TS use; Level 3: topical treatment (high potency) including TS ('strongest' and 'very strong' classes, defined according to the JDA 2016 guidelines [2]); Level 2: topical treatment (low-to-medium potency) including TS ('strong', 'medium' and 'weak' classes, defined according to the JDA 2016 guidelines [2]); Level 1: treated with drugs other than cyclosporine A, OS, or TS; Level 0: not treated with studied treatments.

JDA, Japanese Dermatological Association; OS, oral steroids; SD, standard deviation; TS, topical steroids.

Supplementary Table S3. Treatments received and prescribing patterns of steroids in individuals with prurigo, prurigo nodularis (with or without atopic diathesis, and with and without atopic dermatitis) or prurigo chronica multiformis.

**Treatment**	**Prurigo N=8,588**	**PN N=1,946**				**PCM N=273**
		With atopic diathesis n=1,454	Without atopic diathesis n=492	With AD n=961	Without AD n=985	
Treatment, n (%)						
Topical steroid	5,809 (67.6)	1,198 (82.4)	323 (65.7)	872 (90.7)	649 (65.9)	212 (77.7)
Local steroid injection	559 (6.5)	124 (8.5)	26 (5.3)	69 (7.2)	81 (8.2)	17 (6.2)
Oral antihistamine	5,343 (62.2)	1,105 (76.0)	226 (45.9)	778 (81.0)	553 (56.1)	203 (74.4)
UVB	480 (5.6)	176 (12.1)	11 (2.2)	162 (16.9)	25 (2.5)	23 (8.4)
Oral steroid	1,289 (15.0)	281 (19.3)	42 (8.5)	200 (20.8)	123 (12.5)	59 (21.6)
Cyclosporine	117 (1.4)	42 (2.9)	0	41 (4.3)	1 (0.1)	8 (2.9)
Topical antipruritic	467 (5.4)	85 (5.8)	23 (4.7)	64 (6.7)	44 (4.5)	17 (6.2)
Moisturizer	3,766 (43.9)	886 (60.9)	160 (32.5)	720 (74.9)	326 (33.1)	144 (52.7)
Topical vitamin D3 analogs	159 (1.9)	27 (1.9)	9 (1.8)	21 (2.2)	15 (1.5)	4 (1.5)
Dupilumab	45 (0.5)	20 (1.4)	0	20 (2.1)	0	2 (0.7)
Topical JAK inhibitor	370 (4.3)	125 (8.6)	0	125 (13.0)	0	9 (3.3)
Oral JAK inhibitor	2 (0.02)	1 (0.07)	0	1 (0.1)	0	0
Topical tacrolimus	717 (8.3)	260 (17.9)	0	260 (27.1)	0	19 (7.0)
Kampo	282 (3.3)	75 (5.2)	11 (2.2)	53 (5.5)	33 (3.4)	3 (1.1)
Topical capsaicin	8 (0.1)	1 (0.1)	0	0	1 (0.1)	1 (0.4)
Neurotropin[a]	197 (2.3)	34 (2.3)	7 (1.4)	22 (2.3)	19 (1.9)	9 (3.3)

Gabapentin, pregabalin	193 (2.2)	26 (1.8)	7 (1.4)	19 (2.0)	14 (1.4)	7 (2.6)
Thalidomide	1 (0.01)	0	0	0	0	0
Psychotropic	1,006 (11.7)	178 (12.2)	49 (10.0)	112 (11.7)	115 (11.7)	26 (9.5)
Nalfurafine hydrochloride	4 (0.05)	0	0	0	0	1 (0.4)
Macrolide	984 (11.5)	213 (14.6)	42 (8.5)	128 (13.3)	127 (12.9)	40 (14.7)
Steroid prescribing pattern						
Steroid potency, n (%)[b]	n=5,809	n=1,198	n=323	n=872	n=649	n=212
Strongest	2,361 (40.6)	593 (49.5)	125 (38.7)	454 (52.1)	264 (40.7)	94 (44.3)
Very strong	4, 045 (69.6)	860 (71.8)	218 (67.5)	670 (76.8)	408 (62.9)	172 (81.1)
Strong	2, 612 (45.0)	578 (48.2)	113 (35.0)	445 (51.0)	246 (37.9)	89 (42.0)
Medium	1,881 (32.4)	458 (38.2)	69 (21.4)	378 (43.3)	149 (23.0)	66 (31.1)
Weak	17 (0.3)	6 (0.5)	1 (0.3)	5 (0.6)	2 (0.3)	0
Patch	664 (11.4)	190 (15.9)	51 (15.8)	146 (16.7)	95 (14.6)	13 (6.1)
Cumulative dose, mean ± SD						
Topical steroids, g/year[b]						
Strongest	136.2 ± 212.8	165.2 ± 221.8	104.2 ± 166.9	178.6 ± 226.1	113.2 ± 186.0	196.8 ± 254.5
Very strong	172.8 ± 272.7	243.6 ± 316.7	128.3 ± 248.5	285.1 ± 331.7	113.8 ± 226.3	206.3 ± 277.0
Strong	86.1 ± 173.9	120.5 ± 193.8	55.2 ± 102.0	142.8 ± 208.2	50.1 ± 103.7	52.5 ± 75.5
Medium	45.5 ± 102.6	49.2 ± 99.4	35.7 ± 80.4	54.5 ± 107.0	29.6 ± 62.4	68.4 ± 188.0
Weak	77.0 ± 143.0	55.7 ± 57.2	10.0 ± 0.0	42.9 ± 53.5	64.9 ± 77.6	ND
Local steroid injection, g/year	237.0 ± 377.6	346.9 ± 429.2	153.9 ± 272.9	426.4 ± 455.2	143.6 ± 259.3	300.9 ± 410.9
Oral steroids, mg/year	273.0 ± 516.2	313.1 ± 565.5	319.9 ± 517.3	372.6 ± 637.5	218.5 ± 382.4	312.5 ± 416.8

Prescription duration of oral steroids, days, mean ± SD	46.8 ± 77.1	54.7 ± 81.5	52.5 ± 82.6	62.7 ± 86.8	40.9 ± 70.3	71.2 ± 101.0
Daily dose of oral steroids, mg, mean ± SD	10.1 ± 13.7	9.5 ± 11.4	9.6 ± 9.6	9.7 ± 11.6	9.4 ± 10.4	6.2 ± 4.2
Proportion of days covered with oral steroids, mean ± SD	12.8 ± 21.1	15.0 ± 22.3	14.3 ± 22.6	17.2 ± 23.7	11.2 ± 19.2	19.5 ± 27.6

[a]Extracted fluid from the inflamed skin of rabbits inoculated with vaccinia virus.

[b]Potency of steroids was defined according to the JDA 2016 guidelines.[2]

AD, atopic dermatitis; JAK, Janus kinase; JDA, Japanese Dermatological Association; ND, no data; PCM, prurigo chronica multiformis; PN, prurigo nodularis; SD, standard deviation; UVB, ultraviolet B.

Supplementary Table S4. Healthcare resource utilization in individuals aged >15 years diagnosed with prurigo and included in the cohort analysis.

**Item**	**Prurigo**<br>**N=8,588**
Outpatient visits, n (%)	5,005 (58.3)
Median (Q1–Q3), days/year	4 (2–8)
Range (min, max)	1, 156
Hospitalizations, n (%)	901 (10.5)
Median (Q1–Q3), days/year	6 (3–12)
Range (min, max)	1, 344
Clinical laboratory tests, n (%)	362 (4.2)
Median (Q1–Q3), times/year	1 (1–2)
Range (min, max)	1, 16
Prescriptions for prurigo-associated medications, n (%)	5,924 (69.0)
Median (Q1–Q3), times/year	3 (2–7)
Range (min, max)	1, 62

max, maximum; min, minimum; Q, quartile.

Supplementary Figures

Supplementary Figure S1. Design of the cohort analysis.

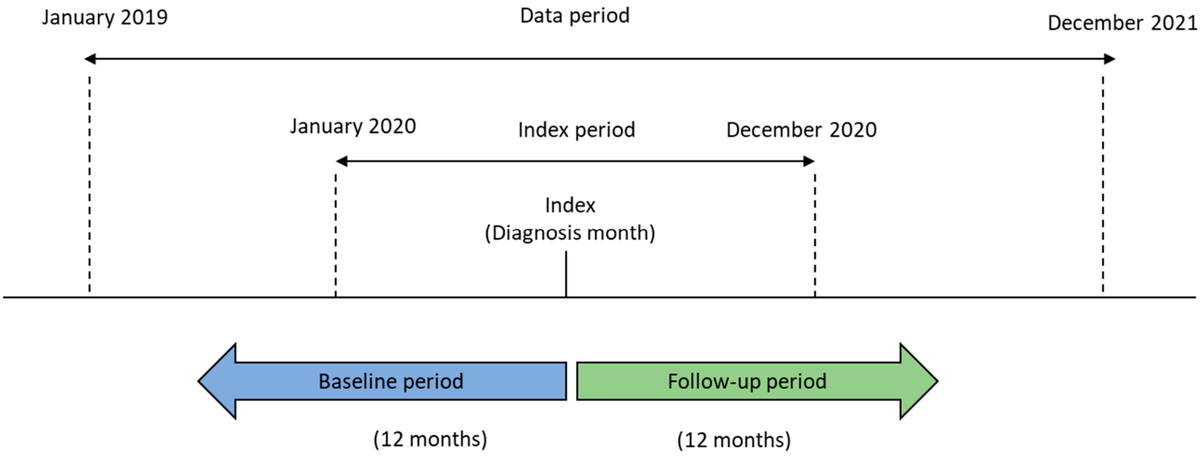

Supplementary Figure S2. Flow of individuals in the cross-sectional analysis. PN, prurigo nodularis.

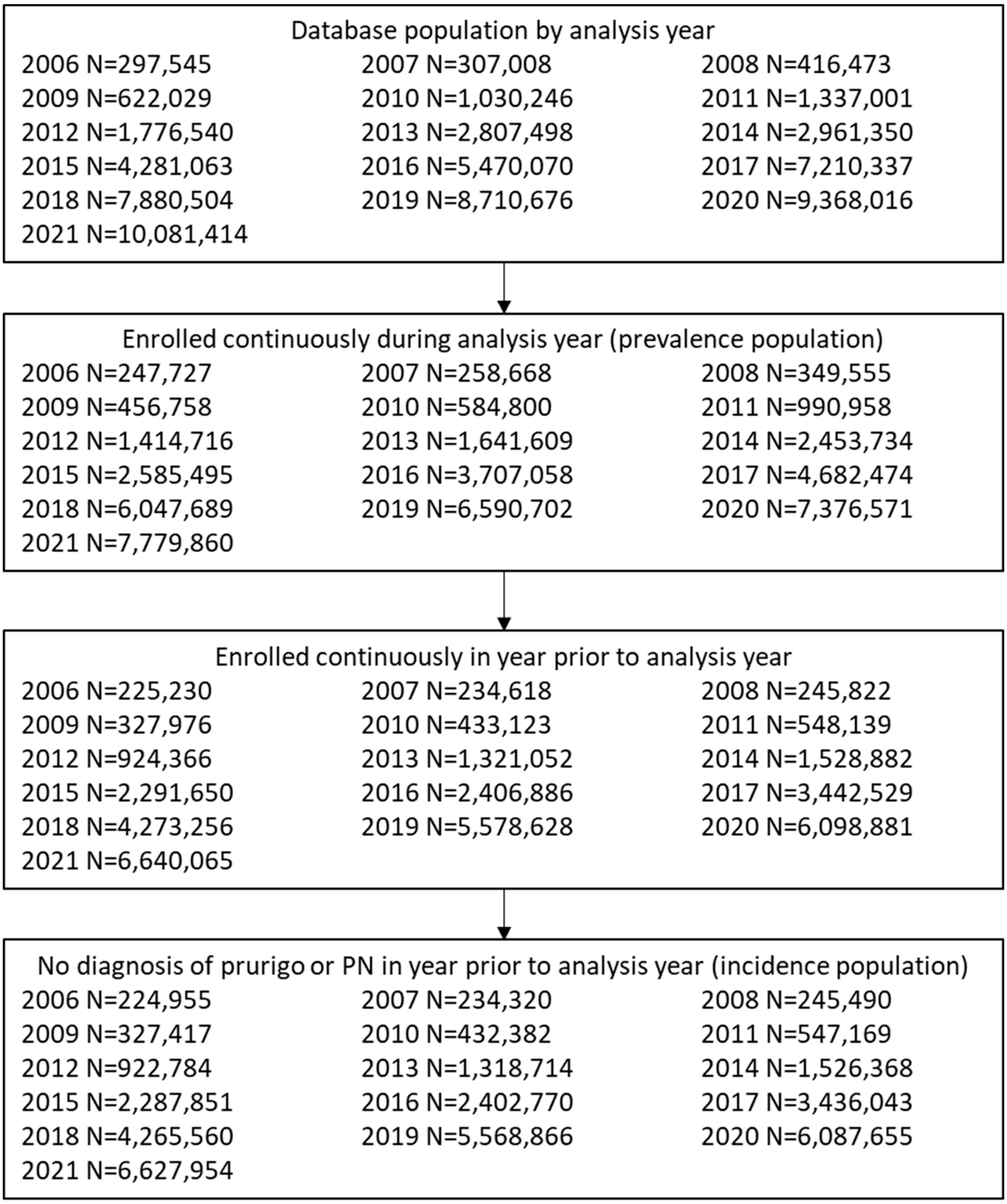

Supplementary Figure S3. Flow of individuals in the cohort analysis. PCM, prurigo chronica multiformis; PN, prurigo nodularis.

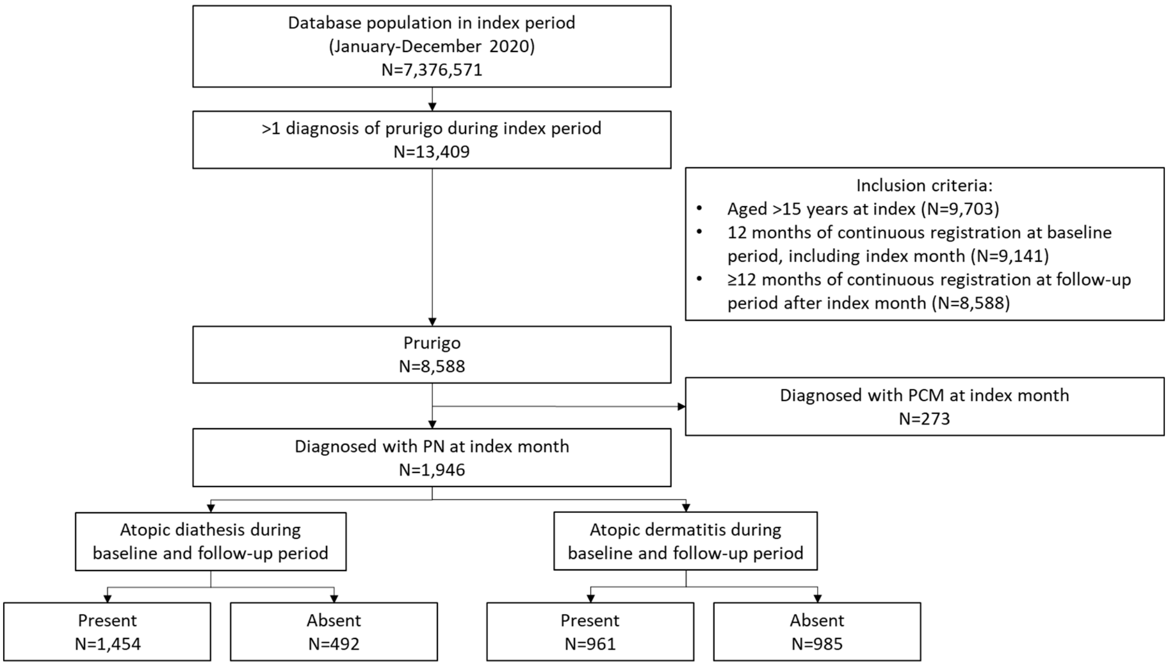

Supplementary Figure S4.(A) Overall prevalence and (B) new onset incidence of prurigo nodularis and prurigo each year from 2006 to 2021 by sex.

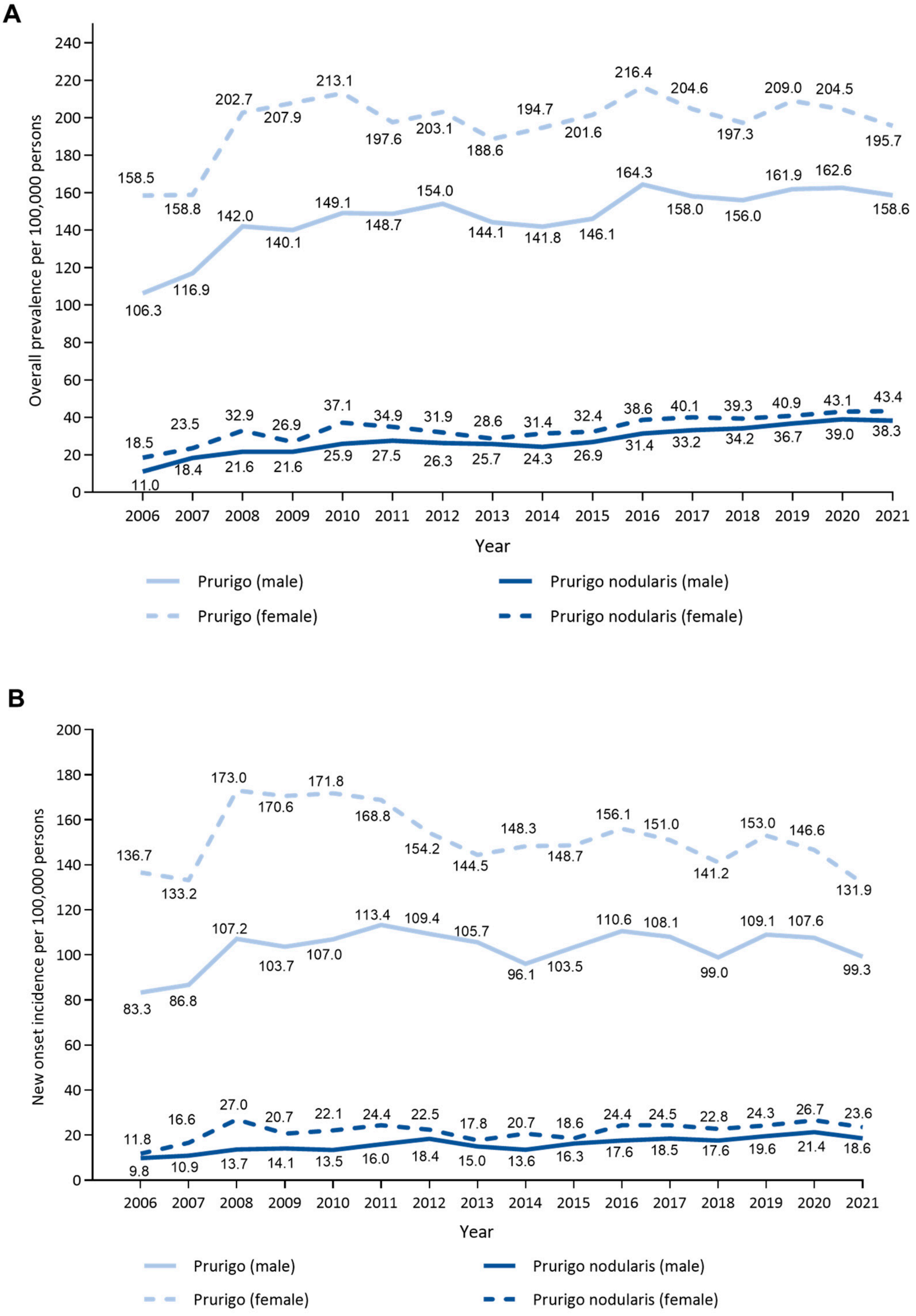

Supplementary Figure S5. (A) Overall prevalence and (B) new onset incidence of prurigo nodularis and prurigo from 2006 to 2021 by presence or absence of atopic diathesis.

A

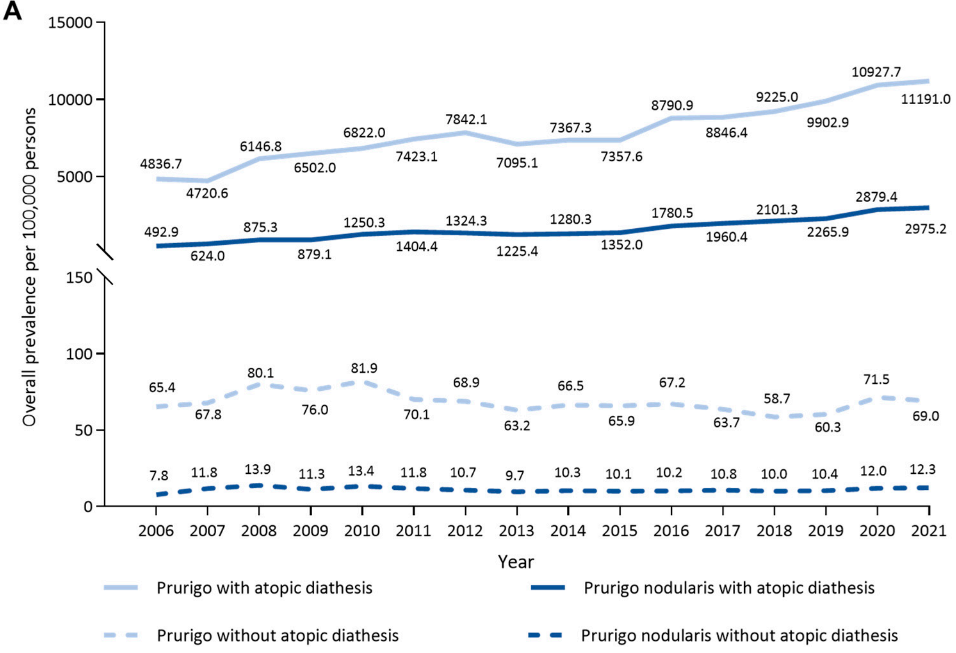

B

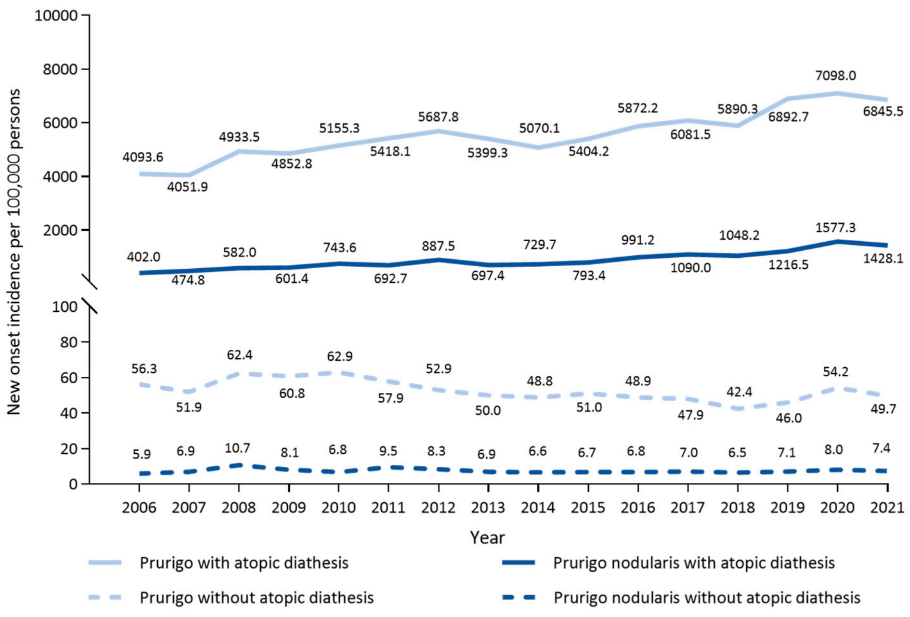

Supplementary Figure S6. (A) Overall prevalence and (B) new onset incidence of prurigo in 2021 by age group.

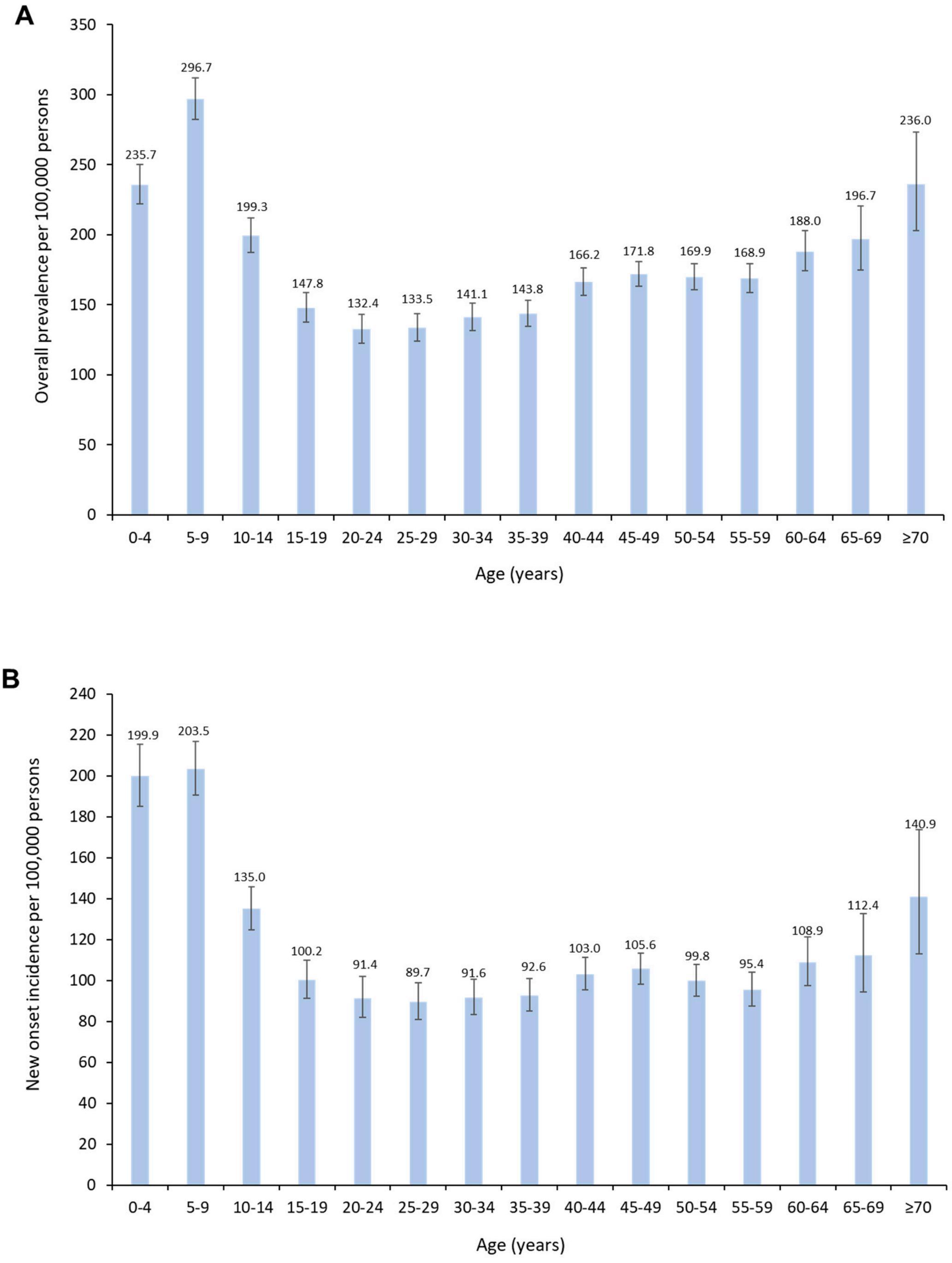

Supplementary Figure S7. (A) Overall prevalence and (B) new onset incidence of individual classifications of prurigo. The overall prevalence and new onset incidence of Hebra's prurigo and papular urticaria were <0.001 in each analysis year.

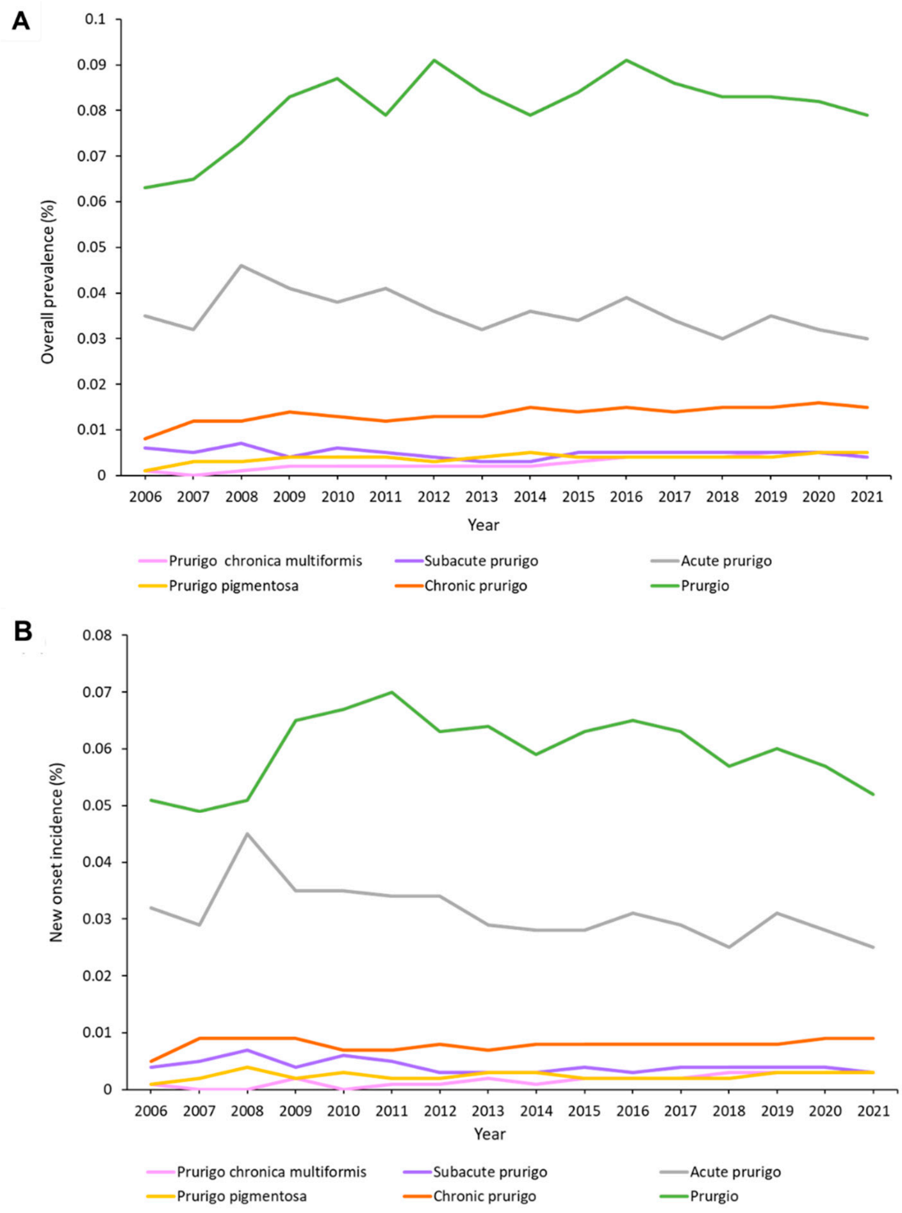

Supplementary Figure S8. Overall prevalence of comorbidities in individuals with prurigo nodularis by absence or presence of atopic dermatitis. Substance abuse was reported in <0.1% of the cohort. AD, atopic dermatitis; COPD, chronic obstructive pulmonary disease; HIV, human immunodeficiency virus.

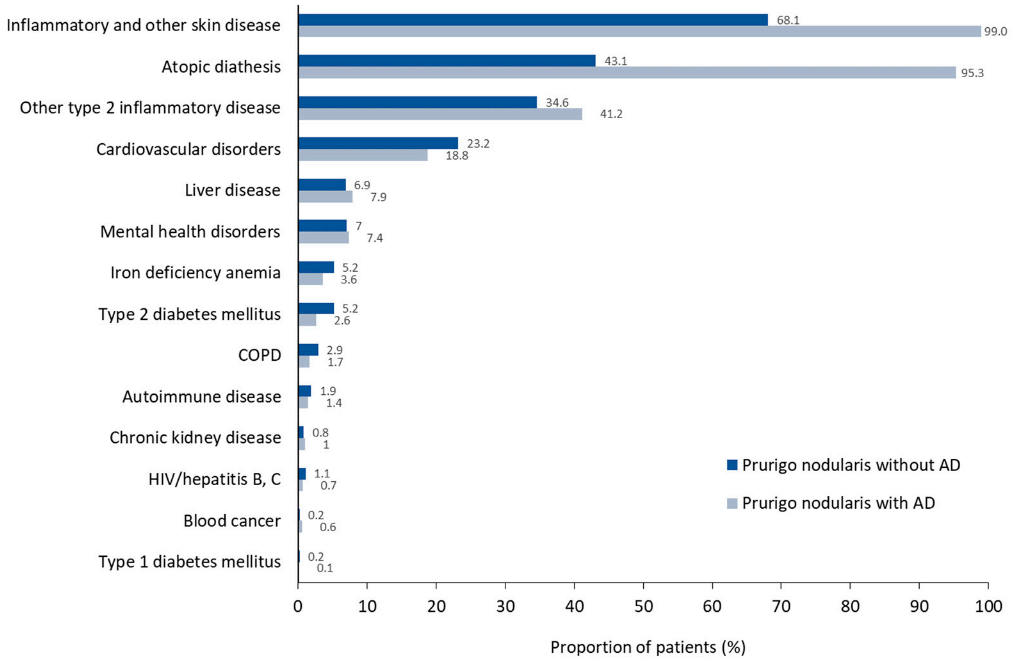

Supplementary Figure S9. Overall prevalence of comorbidities in individuals with prurigo. Substance abuse was reported in <0.1% of the cohort (not shown). COPD, chronic obstructive pulmonary disease; HIV, human immunodeficiency virus.

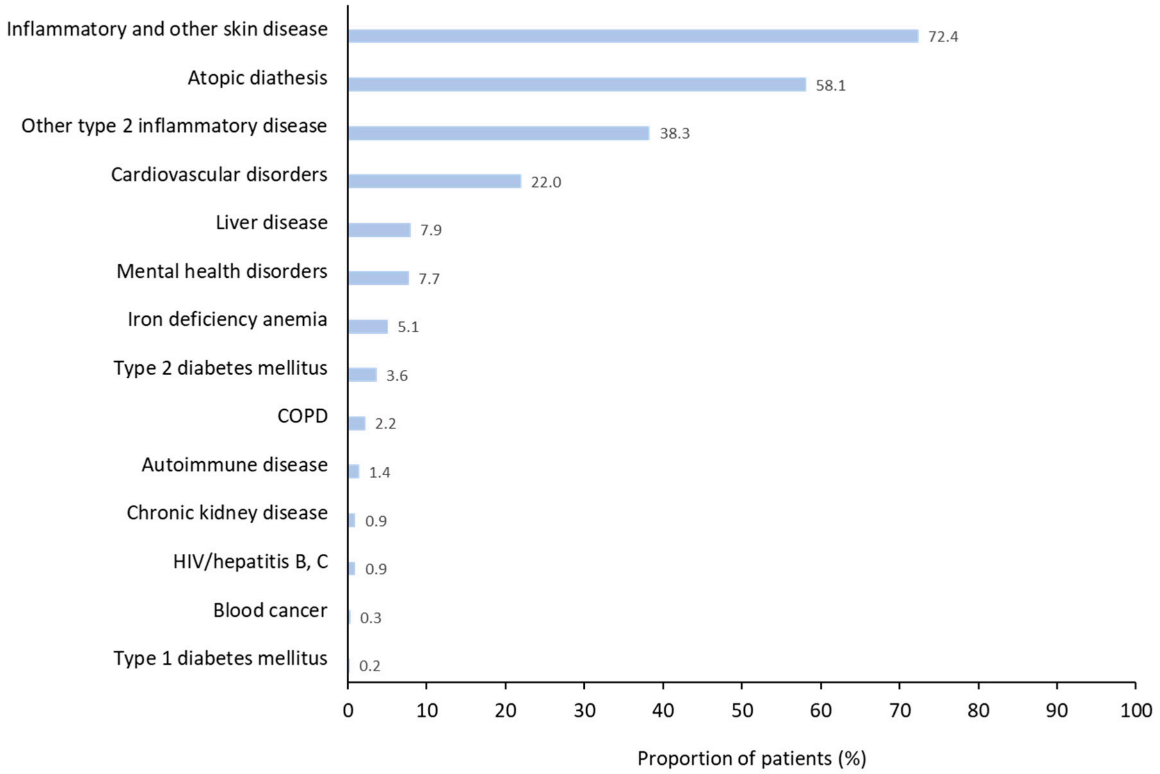

Supplementary Figure S10. Prevalence of comorbid (A) inflammatory and other skin diseases and (B) atopic diathesis diseases in individuals with prurigo nodularis or prurigo. Note that atopic dermatitis, one of the atopic diathesis diseases, is shown in (A). IgA, immunoglobulin A.

A

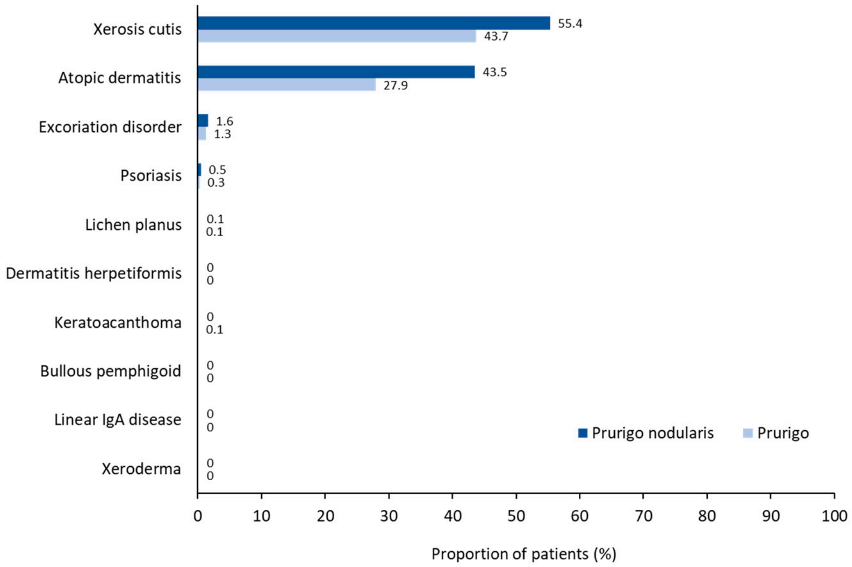

B

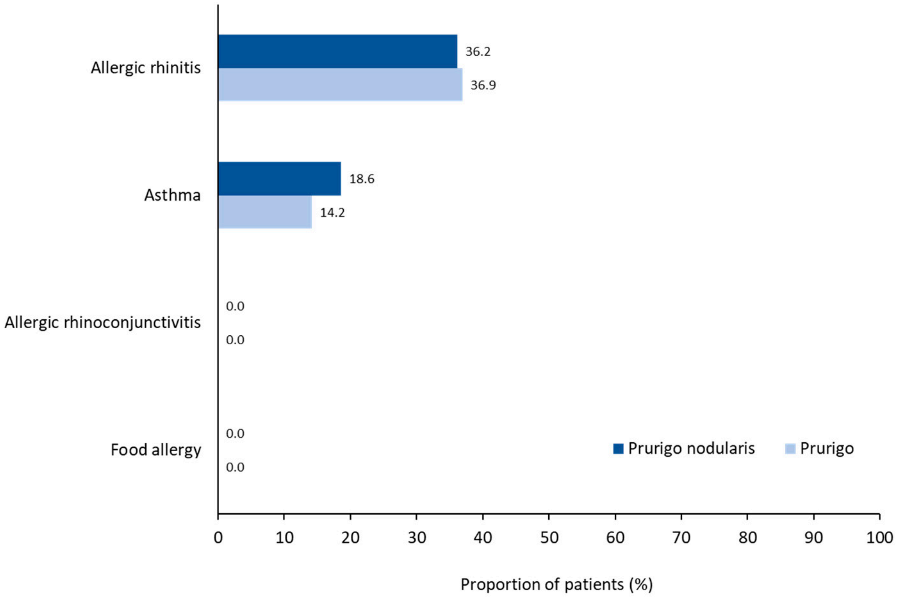

Supplementary Figure S11. Treatment prescribed during the follow-up period in participants with prurigo nodularis by absence or presence of atopic dermatitis. Treatments are categorized based on the Japanese Dermatological Association 2020 guidelines for prurigo.[5] Line 1 (L1) = topical steroid (TS) therapy ± antihistamines ± ultraviolet B phototherapy (with nalfurafine hydrochloride for individuals on hemodialysis, with chronic liver disease, or with other forms of prurigo); L2 = taper TS potency + moisturizer + tacrolimus ointment; L3 = TS therapy + adjunctive therapy (including combined used of local steroid injection, topical heparinoid [occlusive application], vitamin D3 analogs, tacrolimus ointment, antipruritic ointment, capsicum ointment, liquid nitrogen, neurotropin [extracted fluid from the inflamed skin of rabbits inoculated with vaccinia virus], reserpine, gabapentin/pregabalin, or traditional Chinese medicine); L4 = TS therapy + systemic steroid therapy or cyclosporine; or L5 = antibiotics (macrolides), anti-anxiety agents, or thalidomide. The treatments in the grey box at the bottom of the figure are not included in the guidelines. AD, atopic dermatitis; JAK, Janus kinase; UVB, ultraviolet B.

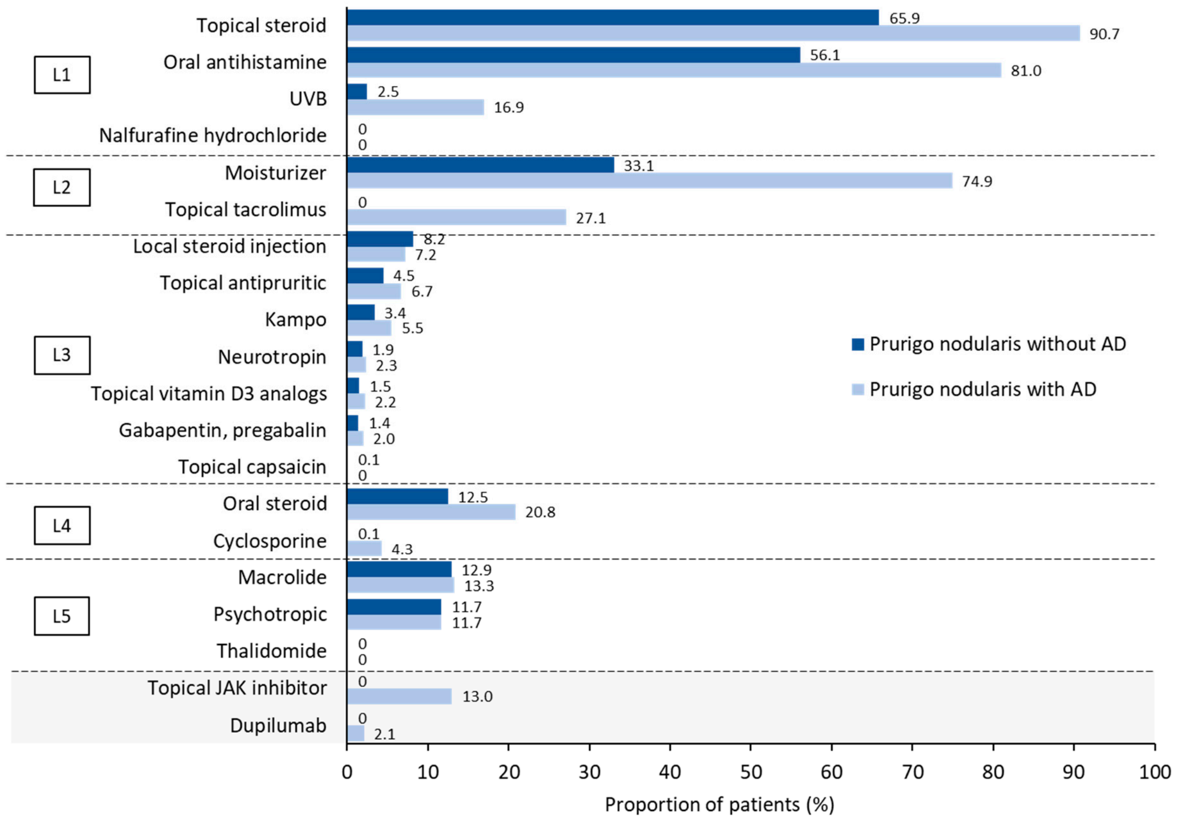

Supplementary Figure S12. Treatment prescribed during the follow-up period in participants with prurigo. Treatments are categorized based on the Japanese Dermatological Association 2020 guidelines for prurigo.[5] Line 1 (L1) = topical steroid (TS) therapy ± antihistamines ± ultraviolet B phototherapy (with nalfurafine hydrochloride for individuals on hemodialysis, with chronic liver disease, or with other forms of prurigo); L2 = taper TS potency + moisturizer + tacrolimus ointment; L3 = TS therapy + adjunctive therapy (including combined used of local steroid injection, topical heparinoid [occlusive application], vitamin D3 analogs, tacrolimus ointment, antipruritic ointment, capsicum ointment, liquid nitrogen, neurotropin [extracted fluid from the inflamed skin of rabbits inoculated with vaccinia virus], reserpine, gabapentin/pregabalin, or traditional Chinese medicine); L4 = TS therapy + systemic steroid therapy or cyclosporine; or L5 = antibiotics (macrolides), anti-anxiety agents, or thalidomide. The treatments in the grey box at the bottom of the figure are not included in the guidelines. JAK, Janus kinase; UVB, ultraviolet B.

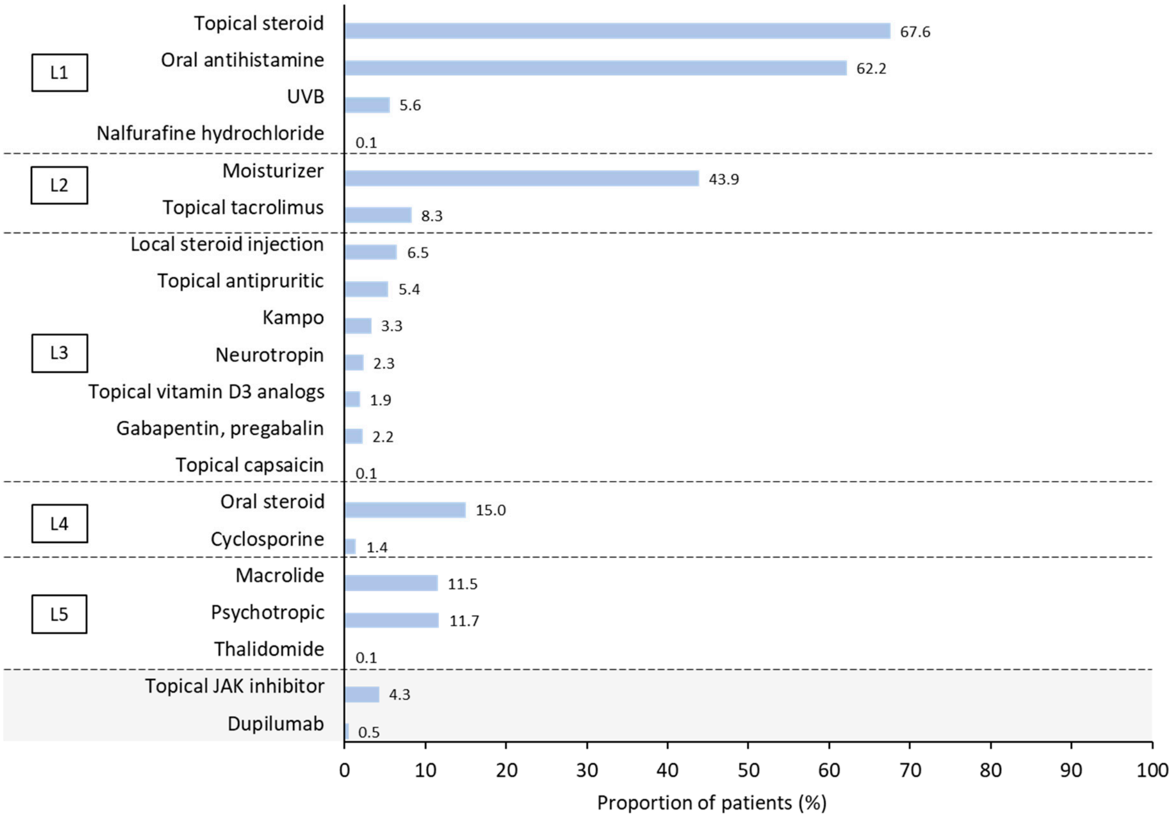

Supplementary Figure S13. Healthcare resource utilization in individuals >15 years diagnosed with prurigo nodularis and included in the cohort analysis who had and did not have atopic diathesis or atopic dermatitis. PN, prurigo nodularis

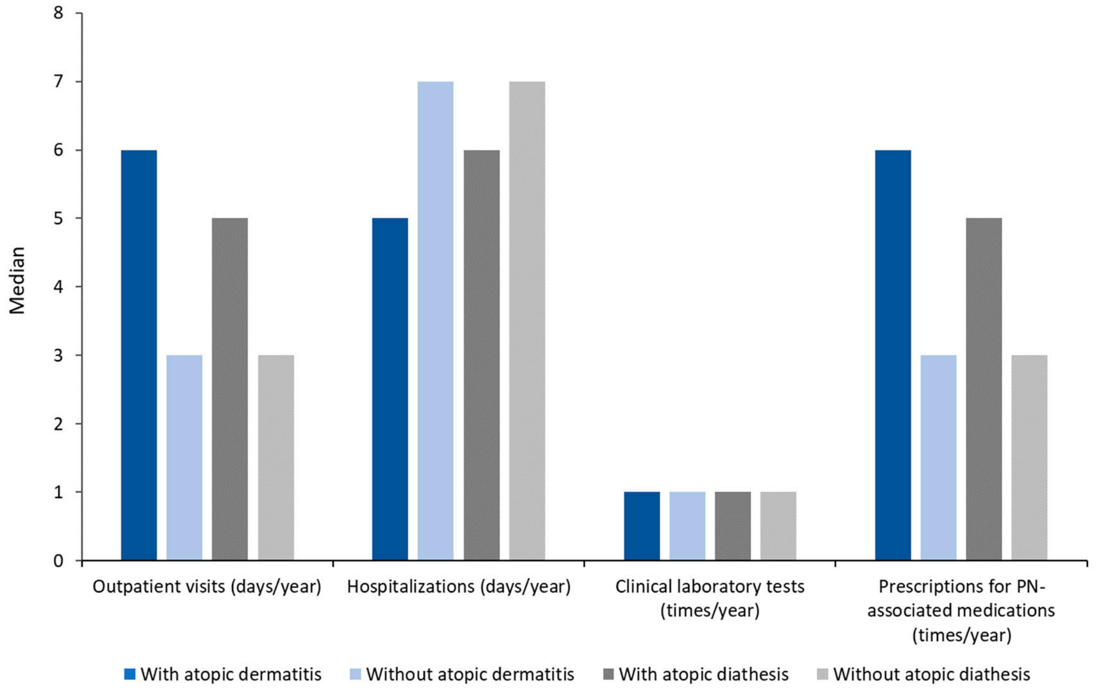

Supplementary Figure S14. Treatment recommendations for prurigo from the Japanese Dermatological Association 2020 guidelines.[5] L, line; UVB, ultraviolet B.

Treatment of prurigo

L1

- Nalfurafine hydrochloride (for patients on hemodialysis, with chronic liver disease, or other forms of prurigo)
- Topical steroid therapy (for ~2 months)
- Antihistamines
- UVB

L2

- Taper potency of topical steroid therapy
- Moisturizer
- Tacrolimus ointment

L3

- Local steroid injection
- Topical therapy with heparinoid (with/without occlusive dressing)
- Vitamin D3 analogs
- Tacrolimus ointment
- Antipruritic ointment
- Capsaicin ointment
- Liquid nitrogen
- Extracted fluid from the inflamed skin of rabbits inoculated with vaccina virus
- Resperine
- Gabapentin/pregabalin
- Traditional Chinese medicine

L4

- Cyclosporine
- Systemic steroid therapy

L5

- Anti-anxiety agents
- Thalidomide
- Antibiotics (macrolides)

References

1. Igarashi A, Fujita H, Arima K, Inoue T, Dorey J, Fukushima A et al. Health-care resource use and current treatment of adult atopic dermatitis patients in Japan: a retrospective claims database analysis. *J Dermatol*. 2019;46:652-661.

2. Saeki H, Nakahara T, Tanaka A, Kabashima K, Sugaya M, Murota H et al. Clinical practice guidelines for the management of atopic dermatitis 2016. *J Dermatol*. 2016;43:1117-1145.

3. Japanese Government Statistics. Population estimates. [cited 2023 21 July]. https://www.e-stat.go.jp/en/stat-search/files?page=1&layout=datalist&toukei=00200524&tstat=000000090001&cycle=7&tclass1=000001011679

4. Ständer S, Pereira MP, Berger T, Zeidler C, Augustin M, Bobko S et al. IFSI-guideline on chronic prurigo including prurigo nodularis. *Itch*. 2020;5:e42.

5. Satoh T, Yokozeki H, Murota H, Tokura Y, Kabashima K, Takamori K et al. 2020 guidelines for the diagnosis and treatment of prurigo. *J Dermatol*. 2021;48:e414-e431.
